# Did the Plague Exterminate the Hellenic People?

**Published:** 1899-12

**Authors:** 


### Did the Plague Exterminate the Hellenic People?

A writer in *Lippincott's Magazine* advances the theory that the Greek nation, of the classic period, was wiped out by the plague in the eighth century; and that, therefore, the modern Greek is not a Greek at all. He says that the classic Hellene, the typical Greek of antiquity as depicted in sculpture and described in poetry, was tall and powerful. So peculiar to his race was his nose that the occasional occurrence of the same kind of nose among modern nations is called Grecian. No other nation, ancient or modern, shows this nose except in rare and sporadic instances. From the Greek sculptures we are led to believe that practically all ancient Greeks had this nose. The ancient Greek was a blonde. His hair was of tightly clinging, reddish curls. His eyes were blue. The modern Greek possesses none of the foregoing physical characteristics save the curly hair, which is dark. Indeed, no Indo-European nation is a more complete contrast to the general conception of the ancient Greek type. Observing these things, it is not strange that a number of German scholars declared that the modern Greek is not a Greek, and that the ancient Greek has passed from the earth, well-nigh as extinct as the dodo. At first though the evidence would seem to prove this theory conclusively. Not only does the modern Greek differ in physical characteristics from the type of ancient Greeks as generally accepted, but there are historical grounds for believing that there is no kinship between the two. In the eighth century a plague devastated Greece. Slavs and Albanians emigrated to fill the depopulated regions. This plague is supposed to have caused the disappearance of the Greek population and the substitution of Slavs and Albanians. —*Indian Lancet.*

